# Variation in an Extreme Weapon: Horn Performance Differences across Rhinoceros Beetle (*Trypoxylus dichotomus*) Populations

**DOI:** 10.3390/insects10100346

**Published:** 2019-10-15

**Authors:** Benjamin Buchalski, Eric Gutierrez, Douglas Emlen, Laura Lavine, Brook Swanson

**Affiliations:** 1 Biology Department, Gonzaga University, Spokane, WA 99258, USA; bbuchalski@zagmail.gonzaga.edu (B.B.); egutierrez@zagmail.gonzaga.edu (E.G.); 2 Division of Biological Sciences, University of Montana, Missoula, MT 59812, USA; doug.emlen@mso.umt.edu; 3 Department of Entomology, Washington State University, Pullman, WA 99164, USA; lavine@wsu.edu

**Keywords:** allometry, sexual selection, force production, armament

## Abstract

Japanese rhinoceros beetle (*Trypoxylus dichotomus*) males have exaggerated head horns that they use as weapons in combat over reproductive opportunities. In these contests, there is an advantage to having a longer horn, and there seems to be little cost to horn exaggeration. However, populations vary in the amount of horn exaggeration across this widespread species. Here, we examine four populations and quantify scaling and functional morphology of the horn. We then measure force production by the horn system in a combat-relevant movement. We find that not only does horn length vary among populations, but allometry of lever mechanics and force production varies in a complex way. For instance, some beetle populations make relatively long horns, but exert relatively low forces. Other populations make shorter horns and produce higher forces during fights. We suggest that this performance variation could be associated with differences in the intensity or type of sexual selection across the species.

## 1. Introduction

Animals produce a vast diversity of weapons that are involved in male–male competition over reproductive access to females [1,2]. A subset of these weapons is exaggerated relative to the body of the organism and scales with positive allometry [3,4,5]. These exaggerated sexually selected structures are more sensitive to nutritional history and the physiological state of the organism than are other body structures and, as a result, they are thought to be honest signals of individual quality [4,6,7,8]. Exaggerated sexually selected weapons therefore may be used as both signals—to assess rival males or to attract choosy females—and as tools of combat [5,9,10,11,12,13,14].

In the rhinoceros beetle mating system, males fight over control of sap sites [9,12,15]. In these fights, the horn is inserted underneath a competitor in order to force the opponent off of the fighting surface [14]. Sap sites function as the primary food source for these beetles. Males control these sites, which females visit to feed, providing males with mating opportunities [9,12,15]. In some populations, males with the largest horns have more copulations than males with shorter horns, suggesting selection for longer horns in males [15,16]. In addition, the cost of horn exaggeration seems to be minimal [17]. Previous studies have found no evidence that exaggerated horns affect flight performance [17,18,19,20], immune function, or the growth of other body parts [21]. However, the relative length of male horns varies among populations [22]. Therefore, this system provides an opportunity to explore the functional consequences of variation in an exaggerated structure.

Here, we measured horn length and other parameters in four populations of rhinoceros beetle. This allowed us to ask how allometry varies across these populations. Then, we asked how force production by the horn weapon system varies within and among populations. Finally, we attempted to explain the relationship between morphology and force production in the context of sexual selection.

## 2. Materials and Methods

Two hundred and nine *Trypoxylus dichotomus* beetles were captured in the field during July and August, 2016 and 2017. The sample included sixty beetles from Puli, Taiwan (24°03′37.9” N, 121°01′12.0” E), sixty from Yakushima, Japan (30°22′49.0” N, 130°38′21.6” E), nineteen from Hokkaido, Japan (44°19′06.9” N, 142°15′31.1” E), and sixty-five from Kyoto, Japan (35°01′07.3” N, 135°30′44.0” E). Variations in sample sizes are due to relative abundance and access to beetle collection sites. Beetles were either captured by hand or by net on sap sites. Additional beetles were captured in traps constructed using a bucket that was covered with a mesh that allowed beetles to pass through but made it difficult for them to escape. The traps were baited with bananas and sake. Traps were hung from tree branches overnight and were collected the following morning.

Morphological measurements were recorded in the field from live beetles using digital calipers (Mitutoyo Corp., Sakado, Japan) to the nearest 0.01 mm. Measurements included horn length, prothorax width, head height, and elytra length. If the horn is modeled as a simple lifting lever, horn length functions as the output lever, the height of the head determines the input lever, which transmits force from the muscles in the prothorax to the horn. Prothorax width was used as an estimate of body size, but we also collected elytra length for some beetles as an additional estimate of body size.

Force production was measured using a Kistler Type 9203 force transducer through a Kistler Type 5995 Charge Amplifier (Kistler Instruments, Winterthur, Switzerland), and recorded with a Vernier LabQuest 2 (Vernier Software and Technology, Beaverton, OR, USA). Beetles were held in place with insect pins placed in the notches between their prothorax and elytra as to not inhibit their force production, but to limit their movement. The force transducer was placed slightly above the tip of the horn attached to a ring stand. The elytra of the beetle were then stimulated to produce a response from the beetles that caused them to push up against the force plate above their horn. This was similar to the motion seen in combat, where beetles lift opponents off of the substrate to dislodge them.

Scaling slopes were estimated using major axis regression in R (https://www.r-project.org). Statistical comparisons were conducted in JMP Pro 13 (SAS Institute, Cary, NC, USA). Measurements were log transformed prior to analysis, and analysis of covariance was used to compare among populations where variables co-varied with body size. Analysis of variance was used to compare among populations where variables did not co-vary with body size.

## 3. Results

**Figure 1:**
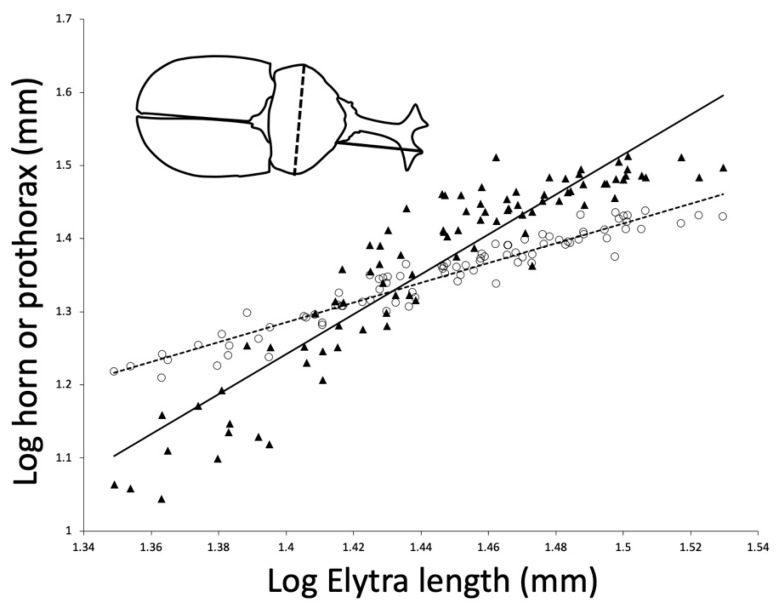
Relationship between horn length, prothorax width, and elytra length in *Trypoxylus dichotomus* males from Kyoto, Honshu, Japan. Both horn length (solid line, major axis slope = 3.15, 95% CI 2.90–3.46) and prothorax width (dashed line, major axis slope = 1.42, 95% CI 1.34–1.51) scale with positive allometry compared to elytra length, although horn length has a steeper slope than prothorax width.

A number of beetle allometry studies use prothorax width as an estimate of body size [14,23,24]. However, because the muscles that lift the horn are in the prothorax, we thought that prothorax size may be under sexual selection and may scale with positive allometry compared to other body size measurements. Therefore, we examined the relationship between elytra length and both prothorax width and horn length in the Kyoto population (Figure 1). We found that prothorax width does scale with positive allometry relative to elytra length (major axis allometric slope = 1.42, 95% CI 1.34–1.51). As expected, horn length also scaled with positive allometry relative to elytra length (major axis allometric slope = 3.15, 95% CI 2.90–3.46).

**Figure 2:**
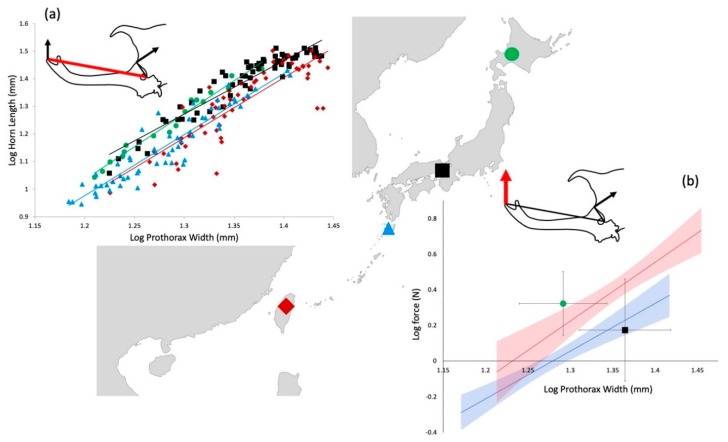
Comparison of scaling of horn length (**a**; red line in drawing) and force production (**b**; red arrow in drawing) among populations of *Trypoxylus dichotomus* males. Both variables are compared across prothorax width. The background is an outline of eastern Asia with sampling locations indicated with colored shapes. Colors also correspond to data in graphs. Green circles = Hokkaido, Japan. Black squares = Kyoto, Honshu, Japan. Blue triangles = Yakushima, Japan. Red diamonds = Puli, Taiwan. In (**a**) slopes are not different, but Hokkaido and Kyoto beetles have relatively longer horns for their body sizes (ANCOVA F_3,200_ = 20.7, *p >* 0.001). In (**b**) Muscles in the prothorax (black arrow in inset drawing) exert force through a simple lever, inserting on the dorsal/caudal surface of the head and rotating the head and horn around a fulcrum just behind the eye. Only Taiwan and Yakushima populations have a relationship between this measured force and body size. For these, a least squares regression line is shown with shaded areas indicating the 95% confidence intervals of slope. Taiwan beetles produce higher forces for a given body size than Yakushima beetles (ANCOVA F_2,117_ = 102.3, *p <* 0.001). Because there is no relationship between force and body size in Kyoto or Hokkaido beetles, populations are indicated as means ± 1 SD (standard deviation) for measured forces and body sizes. Hokkaido beetles produced higher average forces than Kyoto beetles (ANOVA F_1,82_ = 4.6, *p =* 0.03), in spite of having a smaller average body size (ANOVA F_1,82_ = 27.1, *p <* 0.001).

When comparing the relationship between horn length and prothorax width across populations, the allometric slope did not vary (F_3, 200,_ = 1.52, *p =* 0.21; Figure 2a). However, the Kyoto and Hokkaido beetles had significantly longer horns for a given body size, compared to the Taiwan and Yakushima beetles (ANCOVA F_3, 200_ = 20.7, *p >* 0.001; Figure 2a).

The two populations with relatively shorter horns for their body sizes, Taiwan and Yakushima, had positive relationships between body size (prothorax width) and force (Yakushima, F_1,58_ = 47.5, *p <* 0.001, Taiwan, F_1,58_ = 30.7, *p <* 0.001) and between horn length and force (Yakushima, F_1,58_ = 35.8, *p <* 0.001, Taiwan, F_1,58_ = 23.3, *p <* 0.001; Figure 2b). In contrast, the two populations with relatively longer horns, Kyoto and Hokkaido, showed no relationship between body size and force (Kyoto F_61_ = 1.71, *p =* 0.44; Hokkaido F_17_ = 2.48, *p =* 0.14). The Taiwan population produced higher forces for a given body size than the Yakushima population (ANCOVA F_2,117_ = 102.3, *p <* 0.001). Hokkaido beetles produced higher average forces than Kyoto beetles (ANOVA F_1,82_ = 4.6, *p =* 0.03; Figure 2b).

**Figure 3:**
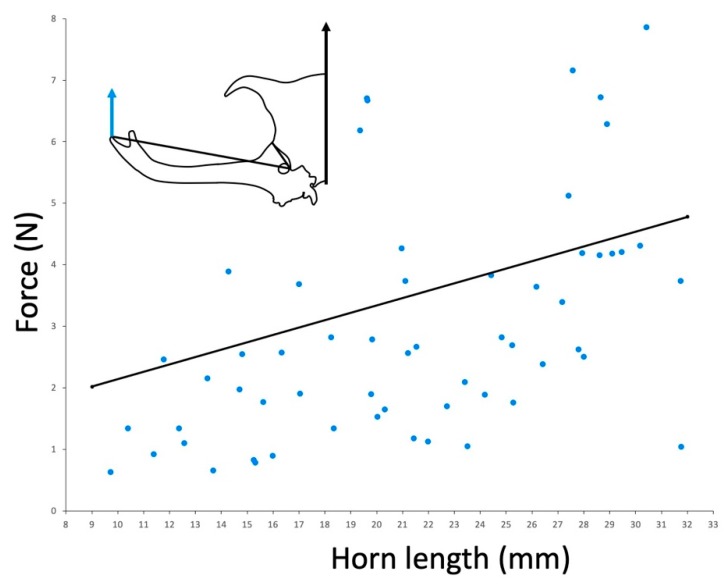
Relationship between measured force (blue arrow in inset) and horn length in the Taiwan population. The black line represents the empirical relationship found between horn length and the force required to pull a beetle off of a tree (black arrow in inset, from [14]).

In the Taiwan population, the force we measured was similar to the force required to dislodge a beetle [14] across a range of body sizes (Figure 3). The force required to dislodge beetles was not measured in other populations.

## 4. Discussion

*T. dichotomus* is widespread with morphologically diverse weapons. Males bear a pronounced pitchfork-like horn with four tines extending vertically from the dorsal surface of the head, as well as a smaller, forward-pointing forked horn extending from the pronotum. Although all populations have the same head and thoracic horn types, the relative size of the head horn varies markedly from population to population, providing an opportunity to understand the conditions under which exaggerated weapons evolve. Our data from one population suggests that exaggeration in beetle horns may sometimes be underestimated compared to other species, as the width of the prothorax, which is used as an estimate of body size in numerous studies [14,23,24], actually scales with positive allometry compared to elytra length. We therefore suggest that the prothorax is part of the sexually selected horn weapon system. However, our analyses continue to use prothorax width as the body size covariate, as to be comparable to other beetle horn literature. Clearly, both prothorax and elytra may vary across individuals and populations for a variety of reasons, and further research will be required to more fully understand the relative scaling of these beetle’s morphology [3,8].

Horn lengths vary widely from male to male in all locations. However, both overall body size and male horn length are phenotypically plastic [16,24,25], responding to the nutritional state and physiological condition of larvae during development. Males modulate weapon growth in response to larval nutrition in a manner consistent with a developmental norm of reaction [16,24]. Consequently, static scaling relationships between horn length and body size approximate the average underlying reaction norms between horn length and nutrition for a population [26,27,28], and population differences in relative horn length likely reflect recent evolutionary shifts in these underlying developmental norms of reaction (for recent reviews of the developmental mechanisms regulating horn growth in this species, see [29,30,31,32].

Here we show that the scaling of horn length varies across our study populations (Figure 2). Beetles from the well-studied population from Kyoto, Japan, tend to have larger body sizes and have exceptionally long horns for those body sizes. Horn length is tightly correlated with copulations and presumably fitness in this population [15]. We found the beetles from Hokkaido in our sample to be relatively smaller in prothorax width, compared to the Kyoto beetles. Although our sample size from this population was relatively small, they also seem to have long horns for their body size (Figure 2).

Taiwanese beetles have relatively large body size and Yakushima beetles are relatively small. Both of these populations have relatively shorter horns for their body size than do males from Kyoto and Hokkaido (Figure 2). There are a number of possible explanations for this observation. It may be that these different horn lengths represent different intensities of sexual selection, or different costs associated with horn exaggeration in these different populations [13].

The real focus of this study was on the functional consequences of this variation in relative horn length. Forces recorded were highly variable across individuals both among and within populations. We think it likely that force producing ability varies widely in populations, however, it is also possible that the amount of motivation or effort varied among individuals. In spite of this variance, we still found patterns and differences in force production among populations (Figure 2).

### 4.1. Force versus Horn Length within Populations

The simple prediction from lever mechanics is that the force a horn can produce should decrease with increasing horn length. However, studies of force generation in other sexually selected weapons suggest that the largest males are often able to compensate for this mechanical disadvantage, for example through increased relative muscle mass, so that weapons maintain, or even increase in effectiveness as they get larger [33,34,35,36].

Our data lend further support to this idea. In *T. dichotomous*, as horn length increases force production can be maintained or even increased. This seems to be due to compensation either in muscle cross-sectional area, or input lever length (the height of the head). Males in the two longest-horned populations (Kyoto, Hokkaido) varied widely in the lifting forces they generated, but this variation was not correlated with horn length.

In the two populations with relatively shorter horns (Taiwan and Yakushima), force exerted at the tip of the horn was positively correlated with horn length. This presumably requires more compensation to not only maintain, but actually increase force with decreasing mechanical advantage of the output lever.

In a set of previous studies, horn force was estimated for the Taiwanese population by measuring the force required to dislodge a beetle from a substrate. When we compare our measurements from this same population, we also find a positive relationship between force and body size, and we find very similar actual values of measured force (Figure 3 [14]). This suggests that the force that beetles produce with their horns and the forces beetles can resist by gripping the substrate may evolve in concert. However, this will have to be examined in other populations to test this hypothesis.

### 4.2. Force versus Horn Length across Populations

Simple lever mechanics again predicts that forces produced by beetles in populations with relatively short horns should be higher than those produced by longer horned populations. Alternatively, a less naive expectation might be that force production would be positively corelated with horn length across populations, therefore indicating correlated evolution between horn length and force compensation. However, the patterns we observed were more complex than this (Figure 2).

First, many males in the longer-horned populations, particularly Kyoto, generated surprisingly weak forces. In fact, the average forces produced by these exceptionally large beetles, were lower than Hokkaido beetles, and lower than similar-sized Taiwanese beetles (Figure 2b). This is in contrast to some individuals in these populations that produced very high forces (some of the highest in the data set). We have no way to tell whether low-force individuals were simply less motivated, or whether they were in fact unable to generate strong forces. Males often fight many dozens of fights in a night (personal observation, [12,15]). This is costly both in the energy required for active tussles, and for the “opportunity cost” that time spent guarding, sparring, and fighting, is time not spent feeding. It may be that males simply “burn out” once they have expended the majority of their stored energy reserves, and that these males cannot generate the same forces that well-fed, “fresh” males can. Alternatively, this pattern of exceptionally long horns and unexceptional average forces could be evidence that in this population the horn is acting more as a signal and a deterrent and is not required to produce high forces to increase male fitness. The observation that there is no relationship between horn length and force production in this population is consistent with this idea. These hypotheses should be testable either in the field or the lab, by comparing the forces generated by individual males when they are well fed versus starved or exhausted from multiple confrontations.

The second caveat worth noting is that our study population with the smallest male body sizes and the shortest relative horn lengths, Yakushima, also had the weakest average forces generated by the males. This is the opposite of what would be predicted by simple lever mechanics, and suggests that males may be experiencing less intense selection for fighting performance in this location, either due to a lower overall density of beetles and therefore fewer contests, or due to weaker net sexual selection on the horn in this population.

The third notable pattern is seen in the Taiwanese beetles. These beetles have relatively large body size and have both relatively high force production and a positive relationship between force and horn length. This is in spite of having a horn length reaction norm that is relatively shorter than the Kyoto or Hokkaido beetles. Force production should be energetically expensive, and this pattern suggests that this population may be under sexual selection that favors horn function in force production over length. However, additional experiments will be needed to explain these observations.

### 4.3. Implications for a Signaling Function of Male Horns

Exaggerated weapons of sexual selection often function as signals, in addition to acting as tools of combat [5]. As these structures get bigger, they are predicted to become both more conspicuous and more honest as indicators of the physiological condition, body size, and/or the genetic quality of their bearer [37]. For this reason, the largest weapons are often used by males to assess the resource holding potential of an opponent and by females as a basis for mate choice [5,11,38,39].

The forked head horn of *T. dichotomus* shares all of the properties of an honest signal of male quality: It is conspicuous; it is hypervariable from male to male, so that even subtle differences in overall body size will be amplified into pronounced—and visible—differences in horn size; and horn growth exhibits ‘heightened conditional expression’ (*sensu* [7]—that is, horn growth is more sensitive to nutrition than is the growth of other body parts [25]. Despite this, it is not yet clear whether the beetles actually use the horn as a signal. Early studies in the lab demonstrate that males can see the horns of an opponent—at least crudely [40], and male fights unfold in a fashion that suggests males do incorporate some form of sequential assessment into their battles (e.g., fights are most likely to escalate when males are evenly matched [9,12,16]. But, to date, no studies have directly tested whether the horns, per se, function as a signal in these confrontations.

One component of resource holding potential (RHP) is the force a male can generate with his horn. Since males use their horns to pry opponents off of the trunk of a tree, males with greater lifting forces should be at a competitive advantage. However, force is not the only element of RHP. For example, longer horns may enable a male to reach his rival before that opponent can reach him (e.g., [41]), and longer horns are correlated with larger overall body size, which is well known to influence contest outcome [42,43]. Nevertheless, we would predict that if horns are a reliable signal of male RHP, then males with longer horns should be able to exert the strongest lifting forces. Interestingly, this prediction holds for the two shorter-horned populations in our study, but it does not hold for the two populations with the longest horns. This is surprising, and merits further study. It is possible that under some circumstances horn length and size may be selected for their signaling function, either as a deterrent or the target of female choice, and that its function as a tool of combat is relatively diminished.

## 5. Conclusions

Rhinoceros beetles are the charismatic megafauna of the insect world. They provide examples of impressively exaggerated sexually selected traits that vary in important ways across their wide and disconnected range. Here, we find that not only does the length of the horn vary from population to population, but force production varies in both magnitude and allometry. This suggests that there has been a complex response to selection, producing a diverse set of fighting morphologies. Further research is required to understand the ecological and mating system variation that has produced this diversity.
